# Transcriptional programming during cell wall maturation in the expanding Arabidopsis stem

**DOI:** 10.1186/1471-2229-13-14

**Published:** 2013-01-25

**Authors:** Hardy Hall, Brian Ellis

**Affiliations:** 1 Michael Smith Laboratories, University of British Columbia, Vancouver, BC V6T 1Z4, Canada; 2 Currently: Swedish University of Agricultural Sciences (SLU), Umeå, 901 83, Sweden

**Keywords:** Cell wall, Anisotropy, Growth kinematic profiling, Transcriptome, Microarray, Arabidopsis, Inflorescence stem

## Abstract

**Background:**

Plant cell walls are complex dynamic structures that play a vital role in coordinating the directional growth of plant tissues. The rapid elongation of the inflorescence stem in the model plant *Arabidopsis thaliana* is accompanied by radical changes in cell wall structure and chemistry, but analysis of the underlying mechanisms and identification of the genes that are involved has been hampered by difficulties in accurately sampling discrete developmental states along the developing stem.

**Results:**

By creating stem growth kinematic profiles for individual expanding Arabidopsis stems we have been able to harvest and pool developmentally-matched tissue samples, and to use these for comparative analysis of global transcript profiles at four distinct phases of stem growth: the period of elongation rate increase, the point of maximum growth rate, the point of stem growth cessation and the fully matured stem. The resulting profiles identify numerous genes whose expression is affected as the stem tissues pass through these defined growth transitions, including both novel loci and genes identified in earlier studies. Of particular note is the preponderance of highly active genes associated with secondary cell wall deposition in the region of stem growth cessation, and of genes associated with defence and stress responses in the fully mature stem.

**Conclusions:**

The use of growth kinematic profiling to create tissue samples that are accurately positioned along the expansion growth continuum of Arabidopsis inflorescence stems establishes a new standard for transcript profiling analyses of such tissues. The resulting expression profiles identify a substantial number of genes whose expression is correlated for the first time with rapid cell wall extension and subsequent fortification, and thus provide an important new resource for plant biologists interested in gene discovery related to plant biomass accumulation.

## Background

Directional (anisotropic) cell wall expansion is an integral part of most plant developmental processes, where it facilitates the structural changes necessary for proper cell and organ morphogenesis. The initial expansive growth phase, which requires both addition of new extracellular polymers and remodeling of existing components in the primary cell walls, is often succeeded by cell wall thickening and rigidification processes to create secondary cell walls that enhance the structural integrity of the organ, but also curtail further wall extension. These sequential processes require a high degree of dynamic, context-specific coordination of cell wall building, reconstruction and fortification in order to harness the underlying driving force of turgor pressure in a spatially-defined manner (reviewed in [1,2]). Consistent with such developmental complexity, at least one thousand genes in Arabidopsis have been shown to have some association with cell wall synthesis and remodeling [3].

The gene expression patterns associated with cell wall expansion and/or secondary cell wall formation in Arabidopsis have been analyzed in several studies in efforts to identify participating genes and understand the biological roles of their products [4-8]. To specifically address cell expansion processes, for example, transcript profiles have been compared with protein accumulation profiles in Arabidopsis seedling hypocotyls that were undergoing rapid cell elongation without significant cell division [9]. Transcript profiling has also been conducted with *in vitro* cultured Zinnia mesophyll cells [10,11] and Arabidopsis subcultured cells [12] that were induced to trans-differentiate into tracheary element-like cells, a process that is accompanied by deposition of distinctive secondary cell wall thickenings on top of the original primary cell walls. In another approach, large-scale correlation analysis of public microarray data enabled the *in silico* identification of genes whose expression is strongly aligned with expression of specific members of the Arabidopsis cellulose synthase (*CesA*) gene family that are believed to be predominantly involved in either primary or secondary cell wall biogenesis [13].

The Arabidopsis inflorescence stem represents an attractive experimental system for such gene discovery studies since it provides more substantial amounts of tissue for analyses, and its tissue architecture is largely established prior to bolting, which means that stem expansion is primarily the product of cell elongation, rather than division. However, the growing stem also presents a continuum of developmental states along the organ as its component cells transition from early anisotropic expansion growth to growth cessation, and finally to cell wall fortification. Integrated with these changes in cell expansion activity are additional changes associated with the differentiation and maturation of the discrete tissues that comprise the stem.

In order to accurately monitor the gene expression changes, or transcriptional programming, that accompany these various stem growth transitions, it is essential to sample and pool stem tissues that are verifiably associated with specific stages of development. A number of inflorescence stem profiling studies have attempted to compare the global transcriptional changes occurring between specific developmental stages [4,5,7,14], but the experimental strategies employed have typically compared tissues from visually selected regions of multiple plants, and have operated under two untested assumptions: 1) that the pooled plants whose stems are being sampled all have similar developmental proportioning, and 2) that the sampling guidelines for the harvested plants, derived from destructive analysis of a different set of plants, accurately associate features such as appearance of lignification in the interfascicular fibres of the stem [4] with specific developmental growth stages. Contrary to these assumptions, growth kinematic profiling of expanding inflorescence stems of individual Arabidopsis plants has recently demonstrated that stem growth profiles actually vary widely from plant to plant, even within genetically homogeneous populations [15]. As a consequence, data obtained from indirectly selected and harvested stem regions are likely to be relatively poorly correlated with onset of processes involved in cell wall extension or modification events associated with specific growth stages.

In this study, we have applied growth kinematic profiling (GKP) to a series of individual Arabidopsis inflorescence stems as described in Hall and Ellis (2012) [15], and used the resulting growth rate profiles to generate pooled samples of stem tissue that accurately represent four discrete growth stages along the cell wall expansion developmental continuum. Our use of GKP-based sampling was expected to reduce the biological noise associated with indirect sampling strategies used in previous studies, and thus increase the sensitivity (power to detect actual differential expression) and accuracy of transcript profiling. Microarray-based assessment of global transcript abundance in these GKP-matched stem samples then enabled us to generate transcriptomic datasets that can be positioned with confidence within a validated developmental context of cell wall expansion performance (relative elemental growth rates). The resulting gene expression profiles demonstrate the participation of many genes that had earlier been linked to primary or secondary cell wall synthesis, but they also highlight expression changes in a range of unique genes whose role(s) in cell wall maturation or stem expansion have yet to be assessed.

## Results

### Stage-specific transcriptome analysis

**Figure 1:**
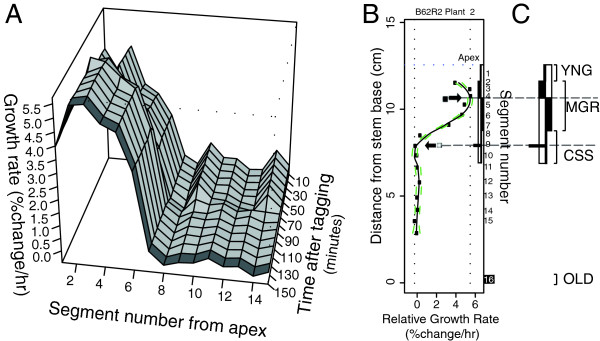
**Representative growth profiling and harvesting. A**) Representative surface plot of relative elongation growth rates (% change per hour, vertical axis) plotted against the number of segments (defined by optical marker tags) from the apex downwards, over the duration of the imaging period in 10 minutes intervals. The darker grey-shaded, nearest profile denotes the last 10-minute interval before harvest, depicted in the greater detail in the right-hand scatterplot. **B)** Corresponding scatter plot of growth rates (% change in length per hour) against distance from the stem base for specific segments. Segments are numbered from the top of the plant downwards in the right-hand margin. The LOWESS regression curve follows the best fit through the growth rate data for this plant over a given 10' interval. Green dotted lines represented 65% confidence intervals for the LOWESS regression curve. Closed-box/arrow indicates the stem position that matches the maximum growth rate of the regression curve (segment 5), plotted as the right-most vertical dotted line, while the open-box/arrow indicates the first position below the top of the stem where the growth rate falls to zero (segment 10). **C)** Harvesting zones for young (YNG), maximum growth-rate (MGR), cessation (CSS), and stem base (OLD) zones based upon LOWESS curve. See methods for description of zone establishment. See Additional file 1: Figure S1 for complete set of 34 growth kinematic profiles.

In order to position transcript profiles accurately within the cell wall expansion continuum, we employed growth kinematic profiling to establish relative elemental growth rates (REGRs) for contiguous stem segments harvested from a series of individual plants [15]. These profiles allowed us to identify three developmental stages for each plant being sampled: 1) an apical region where tissues begin to differentiate, and directional cell growth is initializing (termed ‘young’, or YNG), 2) a region where directional growth of the stem is most rapid (termed 'maximum growth-rate’, or MGR), and 3) a region where elongative growth is finishing (termed 'cessation', or CSS). These stages were therefore each represented by samples consisting of multiple pooled segments, each of which had been harvested from a stem location centered upon a specific GKP-identified growth phase. The tissue selection protocol is outlined in Figure 1A-C, while the growth kinematic profiles for all the plants used in this study are provided in Additional file 1: Figure S1. To facilitate comparison of our data with the results of other transcriptome studies in inflorescence stems, we also harvested a segment (termed 'mature', or OLD) from the base of each of the stems and pooled these for inclusion in our transcript profiling analysis.

To maximize both statistical power and flexibility in analysis, our experiment directly co-hybridized all pair-wise combinations of developmental stage samples (‘complete factorial’ experimental design) and utilized a ‘mixed effects model’ analysis [16] to compare the four developmental stages on the basis of six biological replicates, each pooled from a common set of thirty-four randomly-assigned plants.

### Examination of differential expression between stages

The goal of this study was to identify genes whose expression in the inflorescence stem differs most strongly between different growth stages, since these are expected to represent the loci most actively involved in the accompanying transcriptional reprogramming. The mixed effects model-based analysis of this experiment generates six possible pair-wise comparisons between the four stages, for which the complete statistical analysis is presented in Additional files 2, 3 and 4: Table S1.

For detailed analysis, we focus here on three growth stage comparisons (YNG-MGR, MGR-CSS, and CSS-OLD), and examine the arithmetic differences between the mean (log_2_) signal intensities for each gene. Although as many as 4635 genes are differentially expressed (q-value<0.05) between stages in these comparisons, we have restricted discussion to the forty largest gene expression differences, which are presented as conventional fold-change ratios, together with the log_2_ ratios from which they were derived, and measures of false-discovery-corrected statistical significance of two-sample t-test scores (q-values), for the YNG/MGR (Tables 1 and 2), MGR/CSS (Tables 3 and 4), and CSS/OLD (Tables 5 and 6) comparisons. Since we have applied stringent filtering criteria (see Table legends), these lists should be predominantly populated by genes whose transcript abundances are being modulated in a radical fashion during each associated growth transition. To look for potential functional relationships among these short-listed, up- and down-regulated genes, their annotations, gene ontology (GO) assignments and possible promoter motif enrichment were examined. GO term enrichment was determined relative to whole-genome averages using the ‘AtCoeCis’ web-tool [17], which reports enrichment (fold-change), statistical significance (p-value), and the proportion of the genes in each short-list that have been assigned that specific GO term ('score') (Additional file 5: Table S2, Additional file 6: Table S3, Additional file 7: Table S4).

**Table 1:** Twenty most differentially expressed genes with higher expression in YNG stage relative to MGR stage

**Accession**	**Gene annotation**^**1**^	**YNG/MGR Fold-change**^**2**^	**q-value**^**3**^
AT1G07930	elongation factor 1-alpha / EF-1-alpha	22.5	4.1E-02
AT5G25754	unknown protein	19.2	4.7E-02
AT1G11520	spliceosome associated protein-related	18.9	3.4E-02
AT1G07940	elongation factor 1-alpha / EF-1-alpha	18.9	4.1E-02
AT5G22430	unknown protein	17.6	2.2E-02
AT1G75240	ARABIDOPSIS THALIANA HOMEOBOX PROTEIN 33 (AtHB33)	16.2	1.4E-02
AT4G14080	MATERNAL EFFECT EMBRYO ARREST 48 (MEE48)	15.4	2.2E-02
AT1G01300	aspartyl protease family protein located in membrane, plant-type cell wall	14.6	4.0E-02
AT2G07739	unknown protein	14.4	4.1E-02
AT3G13470	chaperonin, putative with domain Cpn60/TCP-1 (InterPro:IPR002423)	13.1	2.3E-02
AT4G34850	chalcone and stilbene synthase family protein involved in phenylpropanoid biosynthetic process	13.0	2.5E-02
AT3G17840	RECEPTOR-LIKE KINASE 902 (RLK902)	12.6	3.0E-02
AT1G52030	myrosinase binding protein, putative	12.2	2.2E-02
AT5G15720	GDSL-MOTIF LIPASE 7 (GLIP7)	11.6	3.7E-02
AT2G18020	EMBRYO DEFECTIVE 2296 (EMB2296)	11.5	3.6E-02
AT5G62080	protease inhibitor/seed storage/lipid transfer protein (LTP) family protein	11.4	2.9E-02
AT2G01505	CLAVATA3/ESR-RELATED 16 (CLE16)	10.8	4.3E-02
AT3G45140	LIPOXYGENASE 2 (LOX2)	10.6	1.5E-02
AT1G62950	leucine-rich repeat transmembrane protein kinase	10.4	2.7E-02
AT3G55210	ARABIDOPSIS NAC DOMAIN CONTAINING PROTEIN 63 (anac063)	10.0	3.0E-02
AT4G04720	CPK21	9.9	4.2E-02

^1^Gene descriptions are abbreviated from TAIR10 genome release. Putative functions are stated in lowercase.

^2^Genes ranked according to fold-change values derived from log_2_ ratios of YNG (numerator) and MGR (denominator).

^3^ Derived from false discovery-rate correction of p-values.

**Table 2:** Twenty most differentially expressed genes with higher expression in MGR stage relative to YNG stage

**Accession**	**Gene annotation**^**1**^	**YNG/MGR Fold-change**^**2**^	**q-value**^**3**^
AT3G13520	ARABINOGALACTAN PROTEIN 12 (AGP12)	−6.8	3.1E-02
AT1G80170	putative polygalacturonase (pectinase)	−7.3	1.4E-02
AT1G09540	MYB DOMAIN PROTEIN 61 (MYB61)	−7.4	2.6E-02
AT3G05880	RARE-COLD-INDUCIBLE 2A (RCI2A)	−7.5	1.3E-02
AT1G77330	similar to 1-aminocyclopropane-1-carboxylate oxidase	−7.5	1.9E-02
AT1G72430	Auxin responsive SAUR protein	−8.0	3.2E-02
AT4G23496	SPIRAL1-LIKE5 (SP1L5)	−8.1	4.7E-02
AT4G03205	SOUL heme-binding family protein	−8.1	1.9E-02
AT1G67865	unknown protein	−8.2	3.4E-02
AT4G26320	ARABINOGALACTAN PROTEIN 13 (AGP13)	−8.8	1.9E-02
AT3G19710	BRANCHED-CHAIN AMINOTRANSFERASE4 (BCAT4)	−9.6	1.5E-02
AT5G48560	basic helix-loop-helix (bHLH) family protein	−9.9	4.5E-02
AT3G55240	Overexpression leads to PEL (Pseudo-Etiolation in Light) phenotype	−10.0	1.5E-02
AT1G74670	putative gibberellin-responsive protein (GASA6)	−10.2	3.3E-02
AT4G30270	MERISTEM-5 (MERI5B)	−11.5	1.6E-02
AT1G74660	MINI ZINC FINGER 1 (MIF1)	−12.0	4.9E-03
AT4G29905	unknown protein	−12.9	4.3E-02
AT3G45160	unknown protein	−15.3	1.1E-02
AT5G05960	protease inhibitor/seed storage/lipid transfer protein (LTP) family protein	−24.2	1.5E-02
AT5G42180	peroxidase 64 (PER64) located in plant-type cell wall	−40.0	9.4E-04

^1^Gene descriptions are abbreviated from TAIR10 genome release. Putative functions are stated in lowercase.

^2^Genes ranked according to fold-change values derived from log_2_ ratios of YNG (numerator) and MGR (denominator).

^3^ Derived from false discovery-rate correction of p-values.

**Table 3:** Twenty most differentially expressed genes with higher expression in MGR stage relative to CSS stage

**Accession**	**Gene annotation**^**1**^	**MGR/CSS Fold-change**^**2**^	**q-value**^**3**^
AT1G24020	MLP-LIKE PROTEIN 423 (MLP423)	16.7	2.9E-03
AT5G33370	GDSL-like lipase	5.7	2.3E-02
AT2G02320	PHLOEM PROTEIN 2-B7 (AtPP2-B7)	5.2	6.6E-03
AT2G38540	LIPID TRANSFER PROTEIN 1 (LP1)	5.2	5.0E-05
AT5G24780	VEGETATIVE STORAGE PROTEIN 1 (VSP1)	4.8	3.6E-03
AT2G02850	PLANTACYANIN (ARPN)	3.9	2.4E-03
AT2G33810	SQUAMOSA PROMOTER BINDING PROTEIN-LIKE 3 (SPL3)	3.8	4.2E-02
AT3G04290	LI-TOLERANT LIPASE 1 (LTL1)	3.7	2.4E-03
AT1G55490	chloroplast 60 kDa chaperonin beta subunit	3.6	9.1E-04
AT5G20630	GERMIN 3 (GER3)	3.5	3.8E-04
AT2G39670	radical SAM domain-containing protein	3.5	2.1E-02
AT3G47650	bundle-sheath defective protein 2 family / bsd2 family	3.4	3.2E-02
AT5G15230	GAST1 PROTEIN HOMOLOG 4 (GASA4)	3.4	3.1E-04
AT3G47340	GLUTAMINE-DEPENDENT ASPARAGINE SYNTHETASE (ASN1)	3.2	1.3E-02
AT5G20720	CHAPERONIN 20 (CPN20)	3.2	7.6E-03
AT5G55450	protease inhibitor/lipid transfer protein (LTP) family protein	3.2	3.6E-03
AT5G61170	40S ribosomal protein S19 (RPS19C)	3.1	4.3E-02
AT3G08740	elongation factor P (EF-P) family protein	3.1	2.0E-02
AT3G21410	F-box family protein (FBW1)	3.1	2.6E-02
AT2G02130	LOW-MW CYSTEINE-RICH 68 (LCR68)(PDF2.3)	3.0	3.5E-02

^1^Gene descriptions are abbreviated from TAIR10 genome release. Putative functions are stated in lowercase.

^2^Genes ranked according to fold-change values derived from log_2_ ratios of MGR (numerator) and CSS (denominator).

^3^ Derived from false discovery-rate correction of p-values.

**Table 4:** Twenty most differentially expressed genes with higher expression in CSS stage relative to MGR stage

**Accession**	**Gene annotation**^**1**^	**MGR/CSS Fold-change**^**2**^	**q-value**^**3**^
AT5G25110	CBL-INTERACTING PROTEIN KINASE 25 (CIPK25)(SnRK3.25)	−5.1	1.8E-02
AT2G43050	pectin methylesterase	−5.5	2.8E-03
AT4G30290	XYLOGLUCAN ENDOTRANSGLUCOSYLASE (XTH19)	−5.5	2.8E-04
AT5G59290	UDP-glucuronic acid decarboxylase 3 (UXS3)	−5.6	5.8E-04
AT2G38080	LACCASE 4 (IRX12)	−6.0	4.1E-04
AT2G37090	IRREGULAR XYLEM 9 (IRX9)	−6.1	4.3E-04
AT5G46340	REDUCED WALL ACETYLATION 1 (RWA1)	−6.1	3.0E-03
AT1G03740	S/T protein kinase	−6.1	7.8E-06
AT5G01360	TRICHOME BIREFRINGENCE-LIKE 3 (TBL3)	−6.9	2.3E-03
AT2G28315	Nucleotide/sugar transporter family protein	−7.3	4.0E-03
AT1G22480	plastocyanin-like domain-containing protein	−7.8	3.6E-04
AT5G17420	CESA7(IRX3)	−8.8	1.6E-04
AT3G18660	glucuronic acid substitution of xylan1 (GUX1)	−9.0	5.7E-04
AT4G18780	CESA8 (IRX1)	−9.5	9.4E-04
AT3G16920	CHITINASE-LIKE PROTEIN 2 (CTL2)	−10.1	1.3E-05
AT2G28110	FRAGILE FIBER 8 (FRA8)	−10.3	4.8E-04
AT2G03200	aspartyl protease family protein	−10.4	9.6E-05
AT1G63910	AtMYB103	−11.5	2.3E-03
AT5G44030	CESA4 (IRX5)	−11.5	5.6E-05
AT2G45220	pectin methylesterase	−38.4	1.8E-05

^1^Gene descriptions are abbreviated from TAIR10 genome release. Putative functions are stated in lowercase.

^2^Genes ranked according to fold-change values derived from log_2_ ratios of MGR (numerator) and CSS (denominator).

^3^ Derived from false discovery-rate correction of p-values.

**Table 5:** Twenty most differentially expressed genes with higher expression in CSS stage relative to OLD stage

**Accession**	**Gene annotation**^**1**^	**CSS/OLD Fold-change**^**2**^	**q-value**^**3**^
AT1G12845	unknown protein	10.1	4.1E-02
AT5G20630	GERMIN 3 (GER3)	6.5	1.6E-02
AT3G07010	pectate lyase family protein	6.3	7.8E-03
AT1G72610	GERMIN-LIKE PROTEIN 1 (GER1)	6.2	4.0E-02
AT1G64660	ARABIDOPSIS THALIANA METHIONINE GAMMA-LYASE (ATMGL)	5.7	9.6E-03
AT3G15720	glycoside hydrolase family 28 protein / polygalacturonase (pectinase) family protein	5.3	3.5E-02
AT1G80280	hydrolase, alpha/beta-fold family protein	5.3	3.4E-02
AT1G68600	unknown protein	5.0	4.3E-02
AT5G38430	ribulose bisphosphate carboxylase small chain 1B / RuBisCO small subunit 1B (RBCS-1B) (ATS1B)	4.8	4.3E-02
AT2G39010	PLASMA MEMBRANE INTRINSIC PROTEIN 2E (PIP2E)	4.8	2.2E-02
AT2G38540	LIPID TRANSFER PROTEIN 1 (LTP1)	4.7	2.8E-02
AT3G16240	DELTA TONOPLAST INTEGRAL PROTEIN (DELTA-TIP)	4.7	2.2E-02
AT4G03205	coproporphyrinogen oxidase activity in porphyrin biosynthetic process within chloroplast	4.4	3.9E-02
AT3G48970	copper-binding family protein in metal ion transport	4.3	4.9E-02
AT1G75900	family II extracellular lipase 3 (EXL3), carboxylesterase activity, acyltransferase activity	4.3	1.5E-02
AT2G05790	glycosyl hydrolase family 17 protein	4.2	7.8E-03
AT5G38420	ribulose bisphosphate carboxylase small chain 2B / RuBisCO small subunit 2B (RBCS-2B) (ATS2B)	4.2	4.1E-02
AT1G68560	ALPHA-XYLOSIDASE 1 (XYL1)	4.2	4.1E-02
AT5G22580	unknown protein	4.2	2.8E-02
AT3G12610	DNA-DAMAGE REPAIR/TOLERATION 100 (DRT100)	4.1	2.2E-02

^1^Gene descriptions are abbreviated from TAIR10 genome release. Putative functions are stated in lowercase.

^2^Genes ranked according to fold-change values derived from log_2_ ratios of CSS (numerator) and OLD (denominator).

^3^ Derived from false discovery-rate correction of p-values.

**Table 6:** Twenty most differentially expressed genes with higher expression in OLD stage relative to CSS stage

**Accession**	**Gene annotation**^**1**^	**CSS/OLD Fold-change**^**2**^	**q-value**^**3**^
AT1G21310	EXTENSIN 3 (ATEXT3)	−3.9	4.8E-02
AT5G09840	unknown protein	−4.1	3.7E-02
AT3G54580	proline-rich extensin-like family protein, structural constituent of cell wall	−5.0	4.8E-02
AT5G54230	MYB DOMAIN PROTEIN 49 (MYB49)	−5.0	4.1E-02
AT2G28780	unknown protein	−5.0	2.9E-02
AT1G70830	Bet v I allergen family	−5.2	3.9E-02
AT2G02930	GLUTATHIONE S-TRANSFERASE F3 (ATGSTF3)	−5.4	3.0E-02
AT4G15390	acyl-transferase family protein	−6.1	1.6E-02
AT4G08780	peroxidase, putative	−8.2	4.5E-02
AT1G02930	GLUTATHIONE S-TRANSFERASE 6 (GSTF6)	−8.6	4.3E-02
AT2G36120	DEFECTIVELY ORGANIZED TRIBUTARIES 1 (DOT1)	−8.8	3.5E-02
AT1G19530	unknown protein	−9.7	3.1E-02
AT1G75830	LOW-MOLECULAR-WEIGHT CYSTEINE-RICH 67 (LCR67)	−9.9	1.5E-02
AT2G26020	PLANT DEFENSIN 1.2B (PDF1.2b)	−10.7	4.3E-02
AT5G22490	Wax ester synthase homologue	−11.5	4.5E-02
AT5G44420	PLANT DEFENSIN 1.2 (PDF1.2)	−13.1	3.9E-02
AT2G26010	PLANT DEFENSIN 1.3 (PDF1.3)	−16.2	4.4E-02
AT4G16260	O-glycosyl hydrolase	−17.9	4.9E-02
AT5G44430	PLANT DEFENSIN 1.2C (PDF1.2c)	−19.9	3.6E-02
AT3G56700	FATTY ACID REDUCTASE 6 (FAR6)	−55.4	7.8E-03

^1^Gene descriptions are abbreviated from TAIR10 genome release. Putative functions are stated in lowercase.

^2^Genes ranked according to fold-change values derived from log_2_ ratios of CSS (numerator) and OLD (denominator).

^3^ Derived from false discovery-rate correction of p-values.

To establish the status of the stem transcriptome prior to the period at which the maximal elongation rate has been achieved, gene expression profiling was conducted on the top 1 cm segment of the stem (with flowers removed), and this YNG transcript profile was then compared to the profile generated from segments representing the GKP-identified maximum growth rate (MGR) phase. The forty genes exhibiting either high transcript copy number in the YNG stage sample relative to the MGR stage (positive fold-change values), or in the MGR stage sample relative to the YNG stage (negative fold-change values) are listed in Tables 1 and 2, respectively Among the most up-regulated genes in the YNG-dominant set are two members of the four-member *ELONGATION FACTOR 1-ALPHA* gene family [18] (*EF-1-α* A2 (*At1g07930*) and *EF-1-α* A3 (*At1g07940*)). This association with active protein synthesis is also reflected in the GO term enrichment analysis of this set (Additional file 5: Table S2), which indicates >80-fold enrichment in GO terms containing ‘translation’. Also found within this ‘YNG-up-regulated’ list are genes related to signaling (*RLK902*[19]; *CLE16(CLAVATA3 homologue)*[20]; *LOX2*[21]; *At1g62950*, a LRR protein kinase), as well as transcription factors (ZF-HD class *AtHB33*; *NAC063*). Cell-cell communication mediated by peptides derived from CLE gene products, acting together with cognate receptor kinases, represents part of the elaborate signaling network that helps guide plant development [22]. While known cell wall-associated genes are not notably over-represented within the ‘YNG-up-regulated’ list, one gene encoding a putative glucan endo-β(1→3)-glucosidase (*At4g14080*) [23] is up-regulated 15-fold over the MGR stage.

Although cell wall expansion is expected to be taking place in both the YNG and the MGR stage tissues, genes whose products are uniquely required for rapid expansion should be relatively more highly expressed in the latter. The most strongly differentially up-regulated (~40-fold) gene in the MGR tissues relative to YNG is a peroxidase (*PER64*) that has been previously reported to be up-regulated in stems in response to mechanical load [24]. The peroxidase gene family in Arabidopsis is large, and its members play a number of roles in cellular metabolism, including modulation of reactive oxygen species accumulation [25] and the oxidative coupling of aromatic metabolites such as the monolignols that serve as precursors for the lignin polymer [26,27]. The expression of *PER64* in Arabidopsis has been shown to be concentrated in the protoxylem [28], where lignification of patterned secondary cell wall thickenings contributes to cell wall stabilization during vascular elongation, a spatial specificity that is consistent with the strong *PER64* expression in MGR tissues.

The MGR up-regulated list also contains several genes more directly related to primary cell wall formation and re-modeling, including a xyloglucan endotransglycosylase/hydrolase *MERISTEM-5* (*MERI5B/XTH24*), a putative pectinase (*At1g80170),* two arabinogalactan proteins (*AGP12, AGP13*) and a MYB transcription factor (*MYB61*) that has recently been shown to contribute to both cell wall synthesis and regulation of plant carbon allocation [29-32]. In addition, several genes encoding proteins associated with phytohormone signalling are more highly expressed in the MGR tissues, including a putative ACC oxidase, the GA-responsive *MINI ZINC FINGER 1* (*MIF1*) [33] and another gene *GASA6* (*At1g74670)* reported to be GA-responsive [34].

Comparison of the MGR stage gene expression patterns to those observed at the more mature CSS stage provides another view of those genes that are most relevant to active stem expansion, by contrasting their performance in the rapidly expanding MGR tissues with that seen in the CSS tissues where cell wall expansion has ceased. Interestingly, the list of twenty genes whose expression is ‘Higher in MGR relative to CSS’ (Table 3) is led, not by genes known to be associated with cell wall synthesis or modification, but by *MAJOR LATEX PROTEIN 423* (*MLP423*), a member of the BET V1 class of allergens that exhibits sequence homology to ABA- and stress-responsive proteins from various plant species (EMBL-EBI database information). MLP423 is accompanied by two members of the large (108-member) GDSL-type lipase homologue gene family, and by other genes associated with lipid metabolism/transport, but few, if any, genes known to be directly involved in cell wall synthesis are included. This profile implies that the genes populating the ‘Higher in MGR relative to CSS’ list are primarily those whose expression is relatively strongly reduced as the cells make their transition from rapid anisotropic expansion to maturation.

The ‘Higher in CSS relative to MGR’ gene list (Table 4), on the other hand, would be expected to capture those genes that make a major contribution to the re-programming associated with transition to a phase of cell wall stabilization and rigidification. Consistent with this prediction, this list is dominated by genes associated with formation of non-expanding walls, including all three of the cellulose synthase genes believed to be involved in cellulose microfibril deposition during secondary cell wall biosynthesis (*CESA4/IRX5*, *CESA7/IRX3*, and *CESA8/IRX1*) [14,35-37], and *CHITINASE-LIKE PROTEIN 2* (*CTL2*) [38] whose loss-of-function mutant displays cellulose biosynthesis defects [39]. Also strongly represented are genes required for xylan biosynthesis/modification, including a *UDP-GLUCURONIC ACID DECARBOXYLASE 3/UXS3*[40] that provides UDP-xylose for xylan backbone synthesis, *IRREGULAR XYLEM 9* (*IRX9*) [41] and *FRAGILE FIBER 8* (*FRA8*) [42] whose encoded proteins build and extend the glucuronosylxylan polymer, and two xylan modification genes: a xyloglucan-specific endotransglycosylase/hydrolase 19 (*XTH19*) [43], and *REDUCED WALL ACETYLATION 1 (RWA1)*[44]. Other cell wall modification genes are present, including two pectinesterases (*At2g43050*, *At2g45220*), one of which is the most strongly differentially-expressed gene in the list. The prominence of these pectin de-methylating enzymes in the MGR→CSS transition list is consistent with a current model for plant cell wall rigidification in which a reduction in the levels of pectin methylesterification leads to enhanced calcium ion cross-linking and wall stiffening [45-48].

In addition to genes whose encoded products affect cell wall polysaccharide biosynthesis, the list includes *IRREGULAR XYLEM 12* (*IRX12/LAC4*). Laccases are thought to contribute to polymerization of lignin in secondary walls, and *LAC4* expression has previously been shown to be specific to xylary and interfascicular fibres in the Arabidopsis stem. Lignin deposition is largely unaffected in the *lac4* loss-of-function mutant, but is strongly reduced in the *lac4/lac17* double loss-of-function mutant [49]. It is noteworthy that we observed no significant difference in expression of *LAC17* between the CSS and MGR stages (1.3-fold differential, CSS/MGR). Overall, nine of the twenty genes featured in this list also occur among a set of ‘xylem-specific’ Arabidopsis genes identified through analysis of public datasets [50], consistent with a metabolic commitment in CSS tissues to cell wall rigidification in xylem fibres and tracheary elements once stem expansion ceases.

While growth kinematic data cannot precisely position the base of the stem along the developmental continuum (growth kinematic profiling can only distinguish stem regions on the basis of their rates of expansion), it is clear from previous microscopic analysis [4,51] that the OLD stage tissue displays an advanced phase of organ growth and cell wall maturation in the 10-15 cm tall Columbia plants examined in this study. Based on our present understanding of the stem maturation process, the CSS and OLD samples are expected to contain tissues actively engaged in earlier and later stages of secondary cell wall formation and reinforcement, respectively. Tables 5 and 6 present the twenty genes whose expression is ‘Higher in CSS relative to OLD’ and the twenty genes whose expression is ‘Higher in OLD relative to CSS’, respectively.

Displaying high expression in the CSS relative to OLD samples are *GERMIN-LIKE PROTEIN 3 (GER3/GLP3)*(*At5g20630*) and *GERMIN-LIKE PROTEIN 1 (GER1/GLP1)* (*At1g72610*). *GER3* also appeared in the list of genes more highly expressed in MGR tissues than in CSS (Table 3), indicating that expression of this member of the *GER* gene family follows a steeply declining trajectory during the stem maturation process. While specific developmental roles for GLP1 and 3 have yet to be identified, GER proteins are apoplastic glycoproteins that have been widely associated with plant disease resistance and ROS modulation, particularly in the cereals [52]. Interestingly, another Arabidopsis GER homologue (*GLP10*, *At3G62020*) whose expression was previously found to be highly correlated with secondary cell wall-associated *CESAs* (CesA4, 7 and 8) in regression analysis of public microarray datasets [13], also displayed elevated expression at both the CSS and OLD stages in our study (Additional files 2, 3 and 4: Table S1).

Also more highly expressed at this earlier stage of cell wall maturation are two pectate lyases (polygalacturonases), *At3g07010* and *At3g15720*, previously associated with cell separation [53], and *ALPHA-XYLOSIDASE 1/AXY3* (At1g68560*),* an exoglycosylase that acts specifically on non-fucosylated xyloglucans [54] and is essential for apoplastic xyloglucan modification [55]. Several other up-regulated genes are less clearly linked to cell wall processes, but the functions of their encoded proteins may be related to the over-representation of ‘turgor pressure’ in the GO term enrichment analysis for this gene set (Additional file 7: Table S4).

The list of genes most highly expressed in OLD tissues relative to CSS tissues (Table 6) is particularly striking: six of the eight most highly up-regulated genes encode PLANT DEFENSIN (PDF) proteins, small cysteine-rich peptides homologous to anti-microbial peptides that are widely distributed within the eukaryotes [56]. Since both CSS and OLD tissues were harvested only seconds apart, an artifactual pattern of wounding-induced gene induction is not likely. Instead, it appears that accumulation of the products of such classical “defense” genes may form an integral part of the normal maturation of the inflorescence stem, perhaps reflecting a commitment to protection of these tissues until fertilization and seed dispersal are successfully completed.

Relatively few cell wall-specific genes appear in the ‘higher in OLD than in CSS’ short list, with the exception of *EXTENSIN 3/RSH* and another proline-rich extensin-like family protein. EXT3/RSH plays an essential role in cell wall deposition through formation of EXTENSIN protein scaffolds that cross-link other cell wall constituents, thereby contributing to cell wall rigidification [57,58]. The most up-regulated of all the genes at the OLD stage relative to the CSS stage is the chloroplast-localized *FATTY ACID REDUCTASE 6 (FAR6)*. A similar pattern of elevated FAR6 expression was earlier observed in microarray analysis of epidermal peels from the stem base [59] as well as in stem sections harvested from the base of mature Arabidopsis Col-0 plants [6]. Accompanying *FAR6* in this list of most highly expressed genes is a wax synthase homologue (At5g22490), a co-occurrence pattern consistent with epidermal cells in fully mature stems actively synthesizing both their cuticle polyester network and the associated wax matrix. The modest representation of explicitly cell wall-associated genes in this CSS-to-OLD transition list implies that the CSS and OLD stage tissues share quite similar transcriptional profiles in terms of secondary cell wall formation processes, and that the metabolic commitment to cell wall fortification in stem tissues does not change dramatically after cessation of active elongation.

### Stage-specific, whole-genome co-expression analysis

While differential gene expression datasets contrasting discrete growth stages provide initial insights into the biology underlying specific developmental transitions, potential functional relationships between gene products can also be revealed by considering transcript abundances across all the sampled developmental stages. The underlying rationale is that genes co-expressed at one stage and exhibiting similar association patterns across a broader developmental range may represent a subset of genes involved in specific biological processes.

The ‘mixed effects model’ approach used in this study allowed us to generate developmental stage 'estimates' from two-channel arrays, which can be expressed as mean fold-change values (biological replicates = 6) of transcript abundance at one stage relative to a hypothetical mean value of zero across the entire experiment. It should be noted that these 'estimates' can be computed with the same statistical power as applies to the log_2_ differential expression ratios reported in Tables 1, 2, 3, 4, 5, 6. This treatment provides a more intuitive means of visualizing gene expression trajectories, and provides the basis for formal co-expression analysis. The genes associated with each co-expression set (cluster) are identified in a filterable column within the full-genome dataset (Additional files 2, 3 and 4: Table S1), and their AGI codes are also listed separately in Additional file 8: Table S5 for easier access.

**Figure 2:**
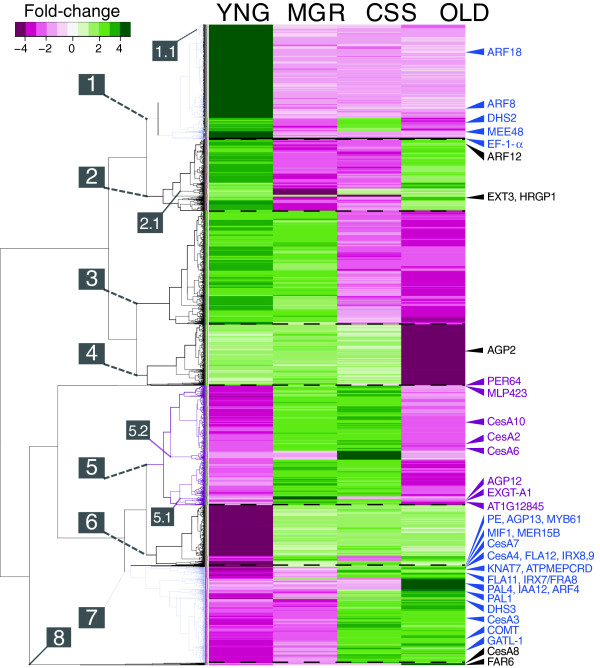
**Hierarchical clustering of 4635 differentially expressed genes (q-value<0.05).** On the basis of relative expression between cell wall expansion stages as outlined in Figure 1; top 1 cm of plant (YNG), maximum growth-rate (MGR), cessation of elongation (CSS) and base of primary stem at rosette (OLD). Inset; 11-level colourimetric fold-change scale. Clusters (1–8) and stage-specific sub-clusters (1.1,2.1,5.1,5.2) are numbered for subsequent examination. The positions of representative genes associated with cell wall processes have been indicated along the right margin (described in ‘Results’ and/or ‘Discussion’). PE=pectin esterase, At2g45220.

Hierarchical divisive clustering was performed on the 4635 genes whose means were most significantly different from the gene-wise mean of all stages (q-value <0.05) (Figure 2), thereby filtering out the great majority (22 294) of genes whose expression displayed little perceptible change during the course of stem elongation. The 4635 genes fall within eight major co-expression clusters that exhibit distinct developmental trajectories. With the exception of cluster 2 (Figure 2), which exhibits elevated expression at only the YNG and OLD stages, genes within the major clusters exhibit a single peak in transcript abundance associated with a discrete developmental stage, accompanied by lower expression in all the sets before and/or after this peak. Each co-expression set (cluster) thus appears to have a uniquely defined developmental window in which the associated genes act, and the clusters are engaged sequentially during elongation and maturation of the inflorescence stem. It is interesting that while most clusters (1–7) contain at least 400+ genes, Cluster 8 (out-group of Clusters 5–7), which contains genes that are up-regulated only at the base of the stem (OLD), is limited to 16 genes.

To test for functional relatedness of the genes populating these clusters, we examined gene ontology enrichment within several sub-clusters that exhibited marked patterns of coordinated up- or down-regulation specific to single developmental stages (Figure 2; sub-clusters 1.1, 2.1, 5.1, 5.2, and Cluster 8). Although box-plotting of the mean 'estimates' for these sub-clusters clearly demonstrates the extent to which these co-expression sets are synchronously up- or down-regulated with respect to the other stages (Additional file 9: Figure S2), gene ontology analysis revealed only modest GO term enrichment within these subsets of co-expressed genes (Additional file 10: Figure S3, Additional file 11: Table S6), indicating that, despite their shared expression pattern, the genes in each sub-cluster do not display obvious functional relatedness.

## Discussion

While gene expression profiling has been applied previously to the expanding inflorescence stem in Arabidopsis, those studies have all suffered from various limitations that impede our ability to accurately align the resulting expression profiles with the developmental state of the tissue being sampled. To address this issue, we have made use of growth kinematic profiling (GKP) to establish, for each plant being sampled, the precise state of growth extension that each stem section represents. Collectively, these sections span the growth and maturation states of the stem, and they also represent a cell wall development continuum. Thus, GKP-guided pooling of sections associated with discrete zones along that continuum (e.g. the point at which extension growth ceases) makes it possible to generate tissue samples whose gene expression profiles can be confidently aligned with specific developmental states.

While the growing stem is a complex organ consisting of multiple tissues, the common denominator across all of these cell types is the coordinated and initially rapid anisotropic expansion of their cell walls along the axis of growth. This expansion ultimately comes to a halt as the walls of some tissues, most notably the vascular tissues and supporting fibres, are reinforced with non-extensible secondary wall layers. Thus, while the biological processes being probed in these samples are not restricted to cell wall expansion/modification, the latter processes can be expected to dominate the broader landscape of transcriptional changes that accompany the maturation of the stem and its component tissues.

### ‘Young’ stage tissue displays a complex transcriptional profile

The YNG stage sampled in this study captures the top 1 cm of the Arabidopsis stem and so encompasses a developmentally complex region containing the shoot apical meristem, and up to twenty short internodes which bore flowers and/or siliques prior to harvest. Since multiple tissue development trajectories are being initiated within YNG samples, it is not surprising that cell wall-forming/modifying processes do not dominate the gene expression profile of YNG stage tissue in comparison with the MGR stage. It is interesting that the up-regulated *MEE48* endo-β(1→3)-glucosidase (Figure 2) was previously characterized as an anther-specific gene whose proposed function involved callose degradation during pollen exine formation [23]. Since all floral tissue had been deliberately removed from the YNG tissue at the time of sampling, MEE48 must play additional roles in development. The prominence of *MEE48* expression in the YNG transcriptome may be related to the importance of callose hydrolysis for the development of new cell plate structures during cytokinesis [60], a process that is actively underway in the apical meristem.

### Rapid tissue extension is associated with a unique transcriptional signature

Both the YNG/MGR comparison and MGR/CSS comparison gene lists provide a perspective on the genes up-regulated in cells undergoing extension growth at their maximum rate (MGR). Genes associated with gibberellic acid (GA)-mediated elongation are prominently expressed at the MGR stage, consistent with the known role of gibberellic acid as an effector of directional cell growth [61]. The redox-associated cysteine-rich signal peptide, *GA-STIMULATED ARABIDOPSIS 4* (*GASA4*) [62], is 3.4-fold up-regulated in MGR relative to CSS (Table 3), while the GA-responsive transcription factor, *MINI ZINC FINGER 1* (*MIF1*) [33], is up-regulated 12-fold in the MGR stage relative to the YNG stage. Loss-of-function at the *MIF1* locus results in unresponsiveness to GA and inflorescence stem dwarfism [33]. *MERISTEM-5* (*MERI5B*, *XTH24;* At4g30270) encodes a Group 2 xyloglucan endotransglycosylase/hydrolase [63], a group of proteins that facilitate the remodelling of hemicellulose to allow cellulose microfibril separation and ‘creep’ during anisotropic cell wall expansion [1]. *MERI5B* expression is also elevated in MGR relative to YNG tissues, and clusters with *MIF1* across all the developmental stages studied (Figure 2). In addition to displaying co-expression, *MIF1* and *MERI5B* co-cluster with the arabinogalactan protein, *AGP13*, and the *MYB61* transcription factor in a set that exhibits peak expression somewhat later, at the onset of cessation (Figure 2, cluster 7).

In contrast to *XTH24*, expression of another *XTH*, *ENDOXYLOGLUCAN TRANSFERASE A1* (*EXGT-A1*), required for normal cell wall expansion [64] is restricted to the MGR stage (Figure 2, cluster 5), where it clusters with another member of the AGP family, *AGP12*, whose expression is >6-fold higher in the MGR stage relative to the YNG stage. An additional five *AGP*s (*AGP14, 21, 22, 24* and *FLA13*) were found to be significantly up-regulated (q-value<0.05) in the MGR stage relative to YNG stage (Additional files 2, 3 and 4: Table S1), suggesting that a suite of AGPs may be contributing to the unique structural dynamics of rapidly expanding cells at the MGR stage.

### ‘Cessation’ stage gene expression is dominated by secondary cell wall processes

The composition of the shortlist of twenty genes most up-regulated at the CSS stage relative to the MGR stage (Table 4) is particularly striking, since thirteen appear to be functionally related to secondary cell wall biosynthesis. It is also notable that the population of this list is completely distinct from those genes whose expression is dominant in CSS tissues relative to OLD tissues, suggesting that these are genes whose expression becomes elevated as cell expansion slows and then remains elevated through ensuing stem maturation.

Central among these cell wall-associated genes are the three cellulose synthases that are essential for secondary cell wall formation [13,35]. CESA8 is thought to belong to the same multi-protein biosynthetic complex as CESA4 and CESA7 [65], which have similar contributions to secondary cell wall synthesis [35], although their relative proportions in the CESA complex remain unknown. In our co-expression analysis, *CESA8* clusters differently from *CESA4* and *CESA7*, primarily due to increasingly elevated expression of *CESA8* in the OLD tissue sample (Figure 2, cluster 8). In contrast, the expression of *CESA4* and *CESA7* (Figure 2, cluster 6) does not change significantly from the CSS to OLD stages. Since the relative stability and turnover rates for the three CESA proteins are unknown, these differences in gene expression do not necessarily conflict with the predicted abundance of their cognate proteins in the plasma membrane. It is also possible that the relative proportions of secondary cell wall-associated CESAs within cellulose synthase complexes do not remain fixed throughout the period of secondary cell wall formation.

By definition, only primary cell walls are capable of expanding [66], and the great majority of this expansion would be occurring above the point of cessation being sampled in this study. We therefore anticipated that cellulose synthase genes associated with primary cell wall formation (notably, *CESA1, 3* and *6*) would be up-regulated in the YNG and MGR stages relative to their expression in the CSS and OLD stages. Instead, CESA1 and 6 are not significantly differentially expressed between pre- and post-cessation stages, and *CESA3* is actually more highly expressed in the CSS and OLD stages than in the YNG and MGR stages (Figure 2, Additional files 2, 3 and 4: Table S1). This is not an isolated example of the behavior of the *CESA3* gene; Ko and Han (2004) [6] had earlier observed elevated *CESA3* expression in the base of fully mature Arabidopsis Col-0 stems (>25 cm in height) relative to the bases of less mature stems (5 and 10 cm in height), while CESA1 and CESA6 expression declined with stem maturation. Ehlting *et al.* (2005) [4] also detected higher levels of *CESA3* expression at the mid-point of 10 cm stems of Landsberg *erecta* (L*er*) plants than in the top 3 cm stem region of those plants. Another study found that, while *AtCESA4, 7* and *8* were up-regulated in mature Col-0 stems, none of the canonical ‘primary cell wall’ CESA genes (*CESA1, 3, 6*) were present in the list of CESAs significantly up-regulated in actively elongating tissues [5], likely due to the persistent expression of CESA1, 3 and 6 at later, non-elongating stages as well. In the present study, *CESA6*, which is generally considered to be important for cellulose deposition in primary cell walls [13], is most highly up-regulated in the MGR and CSS stage tissues (Figure 2, Cluster 5), where it is co-expressed with *CESA2* (previously associated with radial cell wall reinforcement [65]) and with *CESA10*.

Collectively, these data suggest that deployment of particular cellulose synthases in plant cells does not follow a pattern of exclusive association with either actively elongating tissues (i.e. with primary cell wall synthesis) or post-elongation tissues (i.e. secondary cell wall synthesis). Instead, a more diverse co-occurring set of cell wall-forming/modifying processes may recruit distinct sub-sets of CESA and CESA-LIKE family members for specific developmental programming (e.g. intrusive growth of interfascicular fibres).

Another strong indication that the CSS tissue sample accurately captures the transition from primary to secondary cell wall formation is the presence in the ‘higher-in-CSS’ gene lists of a suite of genes specifically associated with accumulation of glucuronylarabinoxylans (‘xylans’), including *XYLOSE SYNTHASE 3*, *FRA8*, *IRX9*, *RWA1*, *XTH19* and *GUX1*[67] (Figure 2, cluster 7). Other xylan-related genes (*XYLOSE SYNTHASE 6*; *XTR4*; *BXL1*; *BXL2*; *EXGT*-*A1*; *XTH18*) are also up-regulated in the CSS tissue (Additional files 2, 3 and 4: Table S1), but failed to qualify for the “top twenty” short-list of genes more highly expressed in CSS than in OLD.

A possible positive regulator of secondary cell wall development, the MYB61 transcription factor gene, is also strongly up-regulated in the CSS stage (Figures 2, S7). MYB61 has been proposed to promote cell wall lignification [31], and more specifically to regulate three cell wall–associated genes encoding the *KNAT7* transcription factor, the lignin biosynthesis enzyme *CAFFEOYL-COA O-METHYLTRANSFERASE 7,* and a pectin methylesterase (*At2g45220*) [29]. While our expression data confirm an association of MYB61 with secondary cell wall formation, such involvement is likely conditioned by other factors since MYB61 activity has also been associated with a wide range of biological processes in plants, including seed coat mucilage production [32], stomatal closure [30], and pleiotropic control of photosynthate partitioning [29].

Arabinogalactan proteins (AGP) form a largely uncharacterized class of proteoglycans that likely play structural and/or signaling roles in cell wall development. Indeed, a number of AGP family members exhibit significant modulation of expression at the onset of secondary cell wall formation (Additional files 2, 3 and 4: Table S1). For example, expression of *AGP18,* a member of a lysine-rich, GPI-anchored sub-family that includes AGP17 and AGP19, is down-regulated at the OLD stage relative to the MGR stage, and the loss-of-function *agp18* mutant possesses shortened inflorescence stems [68], indicating a possible role for AGP18 in promoting cell wall expansion. AGP12 also shows a significant drop in expression coincident with the onset of cessation (Additional files 2, 3 and 4: Tables S1), consistent with a similar functional association.

*FASCICLIN-LIKE 8* (*FLA8*), on other hand, is up-regulated at the OLD stage relative to the MGR stage (Additional files 2, 3 and 4: Table S1). *FLA8/AGP8* belongs to sub-family of AGPs that contain a fasciclin domain and often possess a glycosyl phosphatidyl inositol (GPI) anchor [69]. A poplar homologue of *AtFLA8* was observed to be up-regulated significantly in tension wood, but not in opposite wood, in poplar stems, when compared to its expression in differentiating xylem [70]. *AGP21* also appears significantly up-regulated in the OLD stage relative to the MGR stage (Additional files 2, 3 and 4: Table S1). Interestingly, *AGP21*, similar in sequence to *AGP12* and *AGP14*[71], is down-regulated ~4-fold upon silencing of the transcription factor *PRODUCTION OF ANTHOCYANIN PIGMENT 1* (*PAP1/MYB75*), coincident with increased cell wall thickness in xylary and interfascicular fibres [72]. The members of the large AGP family thus appear to have functionally diverged, as revealed in part through differences in spatiotemporal regulation of their expression [63].

### Protection and fortification are hallmarks of OLD stem tissue

The base of the stem of 10-15 cm Columbia plants (OLD tissue samples) contains highly contrasting tissues, including live, photosynthetically active cells located adjacent to thick-walled, highly lignified fibres of the interfascicular region that are presumably in the advanced stages of programmed cell death.

In general, however, genes related to secondary cell wall synthesis are most active in this region of the lower stem (Figure 2; clusters 2, 6, 7 and 8). Interestingly, *CESA8*, which appears in cluster 8, exhibits its highest level of expression at this stage, as does *XYLOGLUCAN ENDOTRANSGLYCOSYLASE/HYDROLASE 18 (XTH18)*. This expression data is consistent with other results linking xyloglucan deposition with late stages of secondary cell wall synthesis. For instance, incorporation of xyloglucan has been observed to continue in cotton fibres after cessation of wall extension [73], and PttXET16 activity was associated with secondary vasculature of poplar [74].

In the final stages of fibre secondary cell wall maturation, the polysaccharide matrix is typically impregnated with the phenylpropanoid polymer, lignin (reviewed in [75]). Several genes whose products are associated with the shikimic acid and phenylpropanoid pathways, and lignification exhibit corresponding expression patterns within this dataset, although differences in their clustering suggest subtle distinctions in the timing of their expression (Figure 2). For instance, *PAL1*, a member of the *PHENYLALANINE AMMONIA- LYASE (PAL*) gene family whose activity is required for phenylalanine allocation to phenylpropanoid metabolism, appears highly expressed in both the CSS and OLD stages, coincident with up-regulation of *3-DEOXY-D-ARABINO-HEPTULOSONATE 7-PHOSPHATE (DAHP) SYNTHASE 3 (DHS3)*, which regulates the intake of carbon into the shikimate pathway. Maximum expression of *PAL4*, on the other hand, occurs in the OLD stage, suggesting that different PAL family members may be playing distinct roles.

## Conclusion

The large plant-to-plant variation in stem growth kinematic profiles that we identified earlier [15] makes it clear that earlier studies in which stems from multiple plants have been pooled to create biological replicate samples are inevitably compromised in their ability to accurately place cellular changes in an elongative development context. By contrast, the concordance of our GKP-guided gene expression data sets with current knowledge of cell wall biology provides strong evidence of the ability of this approach to capture development stage-specific information. At the same time, those known players in our data sets are accompanied by numerous genes of currently unknown biological function, which makes them high priority candidates for further research into the processes underpinning both plant cell expansion and deposition of the cellulose-rich cell walls that comprise plant biomass.

## Methods

### Plant growth, growth kinematic profiling and sampling

Plant growth and imaging was conducted as described in Hall & Ellis (2012) [15], using applied paper tags as synthetic optical markers for growth kinematic profiling. Tagged and imaged plants were harvested sequentially between 1 and 3 pm (mid-day where daylight cycle occurs between 6 am and 10 pm on a 16hL:8hD regime) at 20-minute intervals. Stem segments (~1 cm) were immediately snap-frozen in liquid nitrogen and deposited into 0.2 mL PCR tubes for −80°C storage. Segments were subsequently pooled on the basis of growth kinematic profiling data (shown in Additional file 1: Figure S1) and experimental design objectives, as outlined in Figure 1.

### RNA processing

Whole stem segments (pooled according to growth kinematic profile equivalence) were homogenized in liquid nitrogen with a pre-cooled mortar and pestle. The frozen powder was then transferred to 1.5 mL microcentrifuge tubes, weighed and combined with TRIzol™ reagent (cat#15596-026, Invitrogen)(1 mL TRIzol per 100 mg tissue), vortexed and incubated at room temperature for 5 minutes. Chloroform (0.2 mL for each 1 mL of TRIzol) was added, vortexed for 15 seconds, incubated for 1 minute at room temperature, and centrifuged at 15000 g for 10 minutes at 4°C. The aqueous phase was transferred to fresh RNAse-free tubes and then combined with an equal volume of isopropanol and incubated 20 minutes on ice. RNA_total_ was pelleted by centrifugation at 15000 g for 10 minutes, and pellets were washed with 1 mL 75% ethanol in RNAse-free water. Following a 5-minute pellet drying phase, pellets were resuspended in 25 μL RNAse-free water and incubated on ice for 1 hour. Each resuspension was treated with 1/10th volume (~2 μL) 10X DNAse I buffer and 1 μL 10X DNAse I (from RNAqueous® Micro kit; cat#AM1931, Ambion) for 20 minutes at 37°C followed by addition of 2 μL DNAse inactivation reagent (also from RNAqueous® Micro kit) and incubated at room temperature for 2 minutes. Samples were centrifuged at 13000 g for 1.5 minutes and the supernatant transferred to RNAse-free tubes and stored at −80°C.

### Reverse transcription and labelling

For each biological replicate, approximately 20 μg RNA_total_ was incubated with 2 ug oligo(dT) primer (cat#18418-012, 0.5 ug/ul, Invitrogen) in a 22.5 μL volume of RNA-primer mix and denatured at 70°C for 10 minutes. Reaction mix was prepared such that each sample contained 9 μL 5X First Strand buffer (supplied with Superscript II, Invitrogen, cat#18064-014), 0.23 μL each of 0.1 mM dATP (cat#10216-108, Invitrogen), dCTP (cat#10217-016, Invitrogen), and dGTP (cat#10218-014,Invitrogen), as well as 0.045 μL dTTP (cat#10219-012, Invitrogen) for a total reaction mix volume of 18.5 μL. This reaction mix was combined with 18.5 μL RNA-primer mix along with 1.5 μL (1.5 moles/μL) of the appropriate Cyanine dye; Cy5-dUTP (cat#45-000-740, Fisher) or Cy3-dUTP (cat#45-000-738, Fisher). After incubation at 42°C for 2 minutes, 1 uL 40U/uL RNAase Inhibitor (cat#10777-019, 40U/ul, Invitrogen) and 1.2 ul 200 U/μL Superscript II (cat#18064-014,200 U/ul, Invitrogen) were added for a final volume of 45 μL which was incubated at 42°C for 2.5 hours, then deactivated with 0.5 M NaH/50 mM EDTA at 65°C for 15 minutes. The reaction was neutralized with 7.1 uL 1 M Tris–HCl (pH7.5). Samples were cleaned of unlabeled probe via centrifugal filtration using Amicon 0.5-Ultra 30 kDa filters (cat#UFC503096, Millipore) prior to array hybridization.

### Array hybridization

For transcript profiling, we employed custom two-channel microarrays spotted with 26 929 70-mer oligonucleotides originally synthesized on the basis of ‘The Arabidopsis Information Resource’ (TAIR) ‘5’ release of the Arabidopsis genome (http://www.Arabidopsis.org), with gene annotations subsequently updated to the current genome release (TAIR10) [76]. The microarray slides were first pre-conditioned by incubating them in Coplin jars with 50°C 2X SSC for 20 minutes, followed by room temperature washes with 0.2X SSC and ddH2O for 5 and 3 minutes, respectively using an Advawash AV400 machine (Advalytix/Beckman-Coulter). Pre-hybridization solution of 1X formamide-based hybridization buffer (pre-warmed to 80°C) from Vial 7 of the 3DNA Array 350 kit (cat#W300130, Genisphere) was then added to the gap between each slide and a pre-placed m-Series lifterslip (cat#48382-251, VWR) within the Slidebooster (Advalytix/Beckman-Coulter) hybridization chamber and subsequently incubated for 1–1.5 hours at 50°C with sonication (power=15, pulse=1 second 'on', 9 seconds 'off'). Slides were then washed in 2X SSC (0.2% SDS) for 15 minutes at 65°C followed by room temperature washes in 2X SSC and 0.2X SSC for 10 minutes each, and centrifuged at 700 rpm until dry in Advatubes (cat# OAX05216, Advalytix/Beckman-Coulter). Equal volumes of labeled Cy3 and Cy5 mixes (12.5 μL each) were combined with 25 μL 2X formamide buffer (Vial 7, 3DNA Array350 kit) and the 50 μL hybridization mix added to the gap between the 42°C pre-warmed slides and the pre-placed m-Series lifter-slips. Slides were then incubated at 42°C for 16–18 hours with sonication (power=15, pulse=1 second 'on', 9 seconds 'off'). Post-hybridization washing was carried out in reduced lighting with 42°C 2X SSC (0.2% SDS) for 15 minutes followed by room temperature washes with 2X SSC and 0.2X SSC for 15 minutes each, then centrifuged until dry at 700 rpm. Slides were stored in a light-proof desiccating chamber until fluorescence scanning.

### Microarray scanning and spot quantification

Hybridized arrays were scanned with a ScanArray Express HT (Perkin-Elmer) scanner and associated software, using 543 nm laser irradiation for Cy3, and 633 m laser for Cy5 fluorescence. Laser power was adjusted for each slide individually within the range of 95-100% such that ~1-2% of spotted probes (presumed positive controls) yielded saturated signals. PMT gain ranged from 60-95%, set for each slide such that fluorescence intensity of sub-grid regions surrounding spots did not exceed 400 (16-bit grayscale). TIFF images of array scans were imported into Imagene (Biodiscovery Software) and grid templates were roughly placed before applying the 'auto-adjust' function to best fit the subgrids on a per-spot basis, allowing spot size variation from 15-20 μm. Median pixel intensities computed from spot regions were used to represent spot intensity in subsequent analyses.

### Microarray data analysis

Data analysis was carried out in the statistical programming environment R (cran.r-project.org/) using custom scripts and contributed packages. To remove local background noise, the mean signal intensity of the dimmest five percent of spots within each of 48 subgrids was subtracted from each array element using a custom script, then variance stabilization normalization (VSN) was applied to each channel to normalize for non-linearity in variance across spot intensities [77] using the function ‘vsn’ (‘vsn’ package, Bioconductor). Normalized intensities were then fit to the mixed effects model [16] using the ‘lme’ function (‘nlme’ package), and all pairwise differential expressions for array elements were computed as the log_2_ intensity difference values between treatment class intensities. Associated measures of significance (p-values relative to null hypothesis, log_2_ difference equals zero) were corrected for false-discovery rate using a custom script based upon standard q-value calculation [78], and ‘estimates’ were computed as the log_2_ intensity difference of each treatment class to the mean of all treatment classes (normalized to zero). Associated measures of significance (p-values relative to null hypothesis of log_2_ difference = 0) were also corrected for false-discovery rate as described above. Raw and output data were exported along with TAIR10 annotations in the supplemental data (Additional files 2, 3 and 4: Table S1). For hierarchical clustering, dissimilarity matrices were computed from filtered datasets using the ‘diana’ function (‘cluster’ package) and rendered as dendrograms using the ‘dendro’ function (‘cluster’ package). Heatmaps were generated using the 'heatmap.2' function ('gplots' package).

### Availability of supporting data

Gene annotations, raw expression data, statistical analysis, mean differentials, mean estimates, and gene categorization for the full genome are provided in Additional files 2, 3 and 4: Table S1, and have been deposited with ArrayExpress following the MIAME conventions [79], as accession E-MEXP-3525.

## Competing interests

The authors declare no competing interests.

## Authors’ contributions

Hardy Hall carried out the microarray experiments and data analyses. Hardy Hall and Brian Ellis both participated in the conception and design of experiments, as well as drafting the manuscript. Both authors have read and approved the final manuscript.

## Supplementary Material

Additional file 1: Figure S1 Surface plots of relative elongation growth rates and LOWESS-predicted growth kinematic profiles (n=34). Plotting as described in Hall & Ellis (2012) except that segments are numbered from bottom upwards for surface plots. Plants arranged by column according to independently grown and observed batches. Click here for file

Additional file 2: Table S1 part 1 Raw, processed data, TAIR10 annotations and clustering information for all gene-specific array elements. See first tab of file for column header descriptions. Click here for file

Additional file 3: Table S1 part 2 Raw, processed data, TAIR10 annotations and clustering information for all gene-specific array elements. See first tab of file for column header descriptions. Click here for file

Additional file 4: Table S1 part 3 Raw, processed data, TAIR10 annotations and clustering information for all gene-specific array elements. See first tab of file for column header descriptions. Click here for file

Additional file 5: Table S2 ATCOECIS reports for enrichment of gene ontology (GO) terms for genes most significantly different (q-value<4.7E-02) between YNG and MGR stages. GO terms appearing more than once in the gene list are shown ranked according to significance of enrichment p-value (<0.05). Only GO terms with two or more genes in the input set and showing enrichment compared to the background frequency (in the full genome) are reported (number of genes indicated in brackets). Score indicates the fraction of input genes annotated with the GO term. 'Term occurrences' column indicates the number of co-occurrences of each GO term in the AtCoeCis results among all expression categories (left-most column). Click here for file

Additional file 6: Table S3 ATCOECIS reports for enrichment of gene ontology (GO) terms for genes most significantly different (q-value<6.72E-02) between MGR and CSS stages. GO terms appearing more than once in the gene list are shown ranked according to significance of enrichment p-value (<0.05). Only GO terms with two or more genes in the input set and showing enrichment compared to the background frequency (in the full genome) are reported (number of genes indicated in brackets).[move to methods; P-values are calculated using the hypergeometric distribution [17]. Score indicates the fraction of input genes annotated with the GO term. 'Term occurrences' column indicates the number of co-occurences of each GO term in the AtCoeCis results among all expression categories (left-most column). Click here for file

Additional file 7: Table S4 ATCOECIS reports for enrichment of gene ontology (GO) terms for genes most significantly different (q-value<4.9E-02) between CSS and OLD stages. GO terms appearing more than once in the gene list are shown ranked according to significance of enrichment p-value (<0.3). Only GO terms with two or more genes in the input set and showing enrichment compared to the background frequency (in the full genome) are reported (number of genes indicated in brackets). Score indicates the fraction of input genes annotated with the GO term. 'Term occurrences' column indicates the number of co-occurences of each GO term in the AtCoeCis results among all expression categories (left-most column). Click here for file

Additional file 8: Table S5 List of Arabidopsis gene index (AGI) codes for each of the genes in the clusters specified in Figure 2. Click here for file

Additional file 9: Figure S2 Boxplots depicting distribution of estimates of relative gene expression (fold-change) of each developmental stage for the sub-clusters identified in Figure 2. Boxes bound upper and lower quartiles, dark horizontal bars denote median values, whiskers represent 95% confidence intervals, circles represent outliers occuring in upper and lower 2.5 percentiles. Cluster 8 is also depicted in Figure 2. Click here for file

Additional file 10: Figure S3 Gene ontology (GO) SLIM term enrichment analysis for clusters depicted in Figure 2. A) Boxplots depicting distribution of term enrichment across all clusters, expressed as fold-change relative to abundance in the full genome, for each of the three GO SLIM categories; 'cellular component', 'molecular function', and 'biological process'. Boxes bound upper and lower quartiles, dark horizontal bars denote median values, whiskers represent 95% confidence intervals, circles represent outliers occuring in upper and lower 2.5 percentiles. B) Barplots exhibiting term enrichment for each cluster in each of the three GO SLIM categories; colour assignment for bars is indicated in Figure 'A'. The number of genes (accessions) included in each cluster is indicated at the base of the 'biological process' barplot. Click here for file

Additional file 11: Table S6 ATCOECIS reports for enrichment of gene ontology (GO) terms for [sub] clusters of genes displaying stage-specific expression. Top 10 most significant over-represented GO terms appearing more than once in the gene list are shown ranked according to p-value (<0.05). Only GO terms with two or more genes in the input set and showing enrichment compared to the background frequency (in the full genome) are reported (number of genes indicated in brackets). Score indicates the fraction of input genes annotated with the GO term. 'Term occurrences' column indicates the number of co-occurrences of each GO term in the AtCoeCis results among all expression categories (left-most column). Click here for file
