# Supplementary material for: Transcriptional programming during cell wall maturation in the expanding Arabidopsis stem
Source: BMC Plant Biol. 2013 Jan 25;13:14. doi: 10.1186/1471-2229-13-14 (PMC3635874; doi:10.1186/1471-2229-13-14)
Supplement: Additional file 9: Figure S2 — Boxplots depicting distribution of estimates of relative gene expression (fold-change) of each developmental stage for the sub-clusters identified in Figure 2. Boxes bound upper and lower quartiles, dark horizontal bars denote median values, whiskers represent 95% confidence intervals, circles represent outliers occuring in upper and lower 2.5 percentiles. Cluster 8 is also depicted in Figure 2. [file 1471-2229-13-14-S9.pdf]

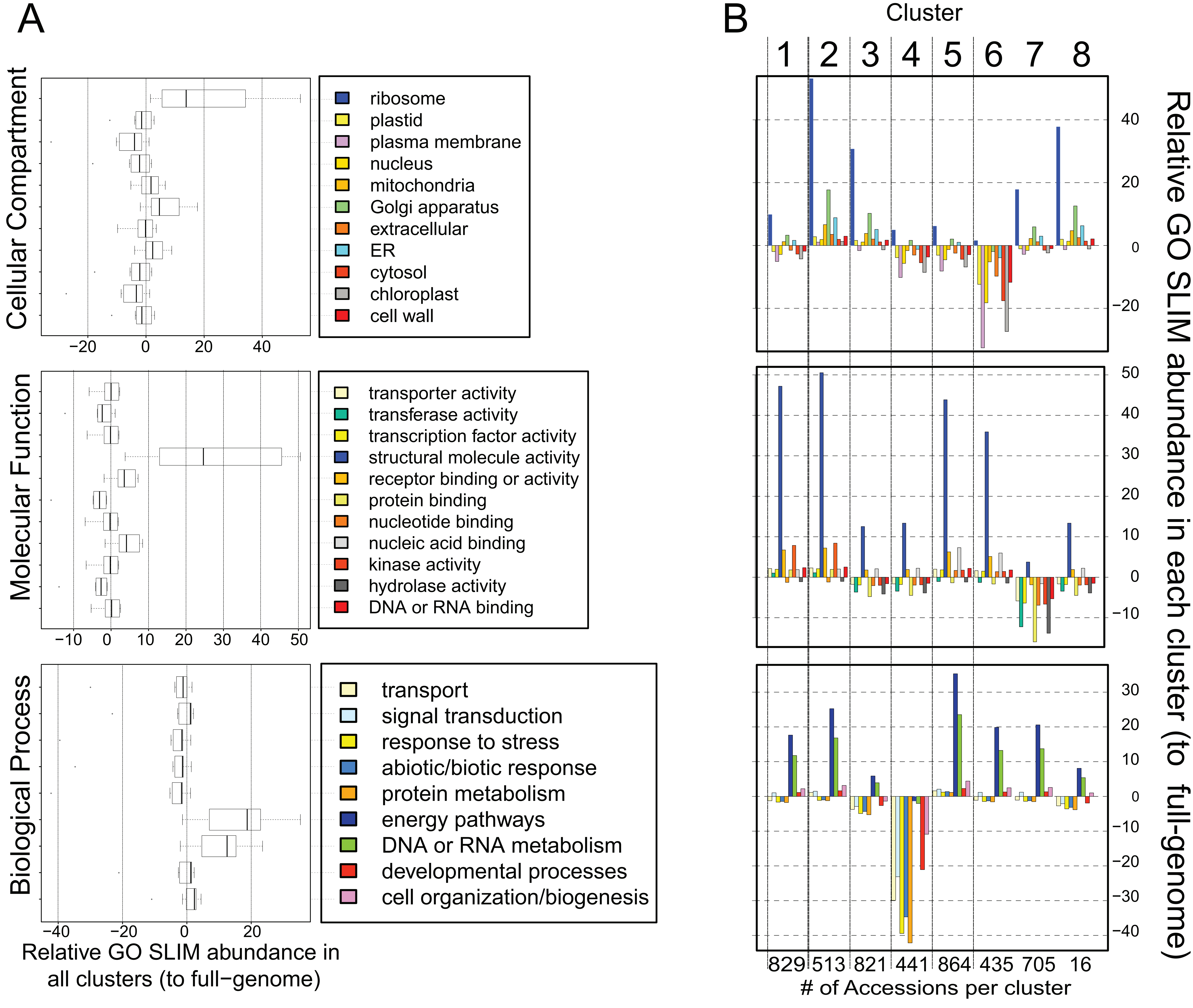

A
B
Cellular Compartment
ribosome
plastid
plasma membrane
nucleus
mitochondria
Golgi apparatus
extracellular
ER
cytosol
chloroplast
cell wall
Molecular Function
transporter activity
transferase activity
transcription factor activity
structural molecule activity
receptor binding or activity
protein binding
nucleotide binding
nucleic acid binding
kinase activity
hydrolase activity
DNA or RNA binding
Biological Process
transport
signal transduction
response to stress
abiotic/biotic response
protein metabolism
energy pathways
DNA or RNA metabolism
developmental processes
cell organization/biogenesis
Relative GO SLIM abundance in all clusters (to full−genome)
Cluster
1 2 3 4 5 6 7 8
829 513 821 441 864 435 705 16
# of Accessions per cluster
Relative GO SLIM abundance in each cluster (to full-genome)
