# Supplementary figures and images for: Transcriptional programming during cell wall maturation in the expanding Arabidopsis stem
Source: BMC Plant Biol. 2013 Jan 25;13:14. doi: 10.1186/1471-2229-13-14 (PMC3635874; doi:10.1186/1471-2229-13-14)

### Sub-Cluster 1.1

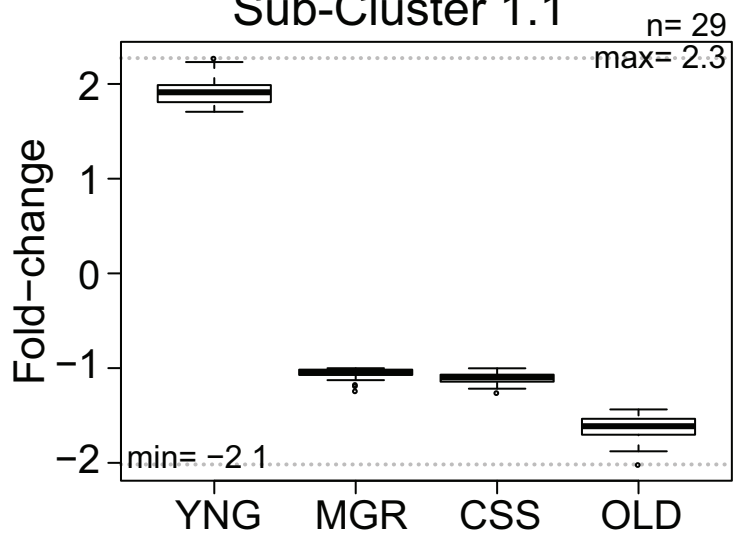

### Sub-Cluster 2.1

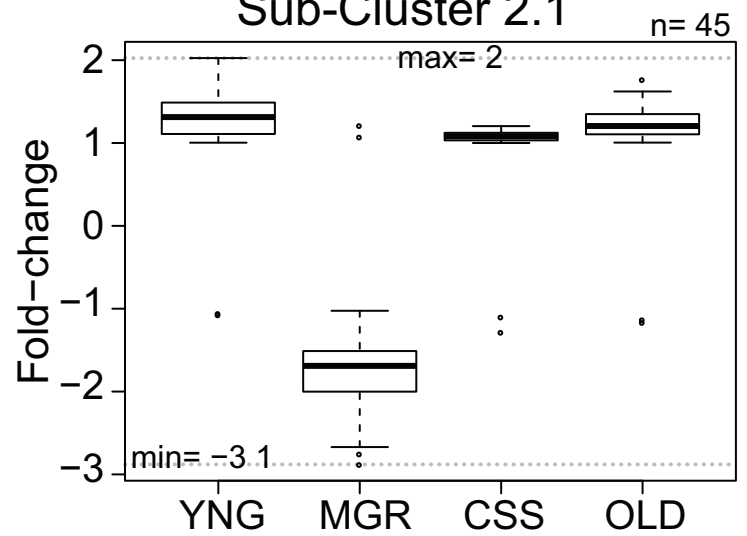

### Sub-Cluster 5.1

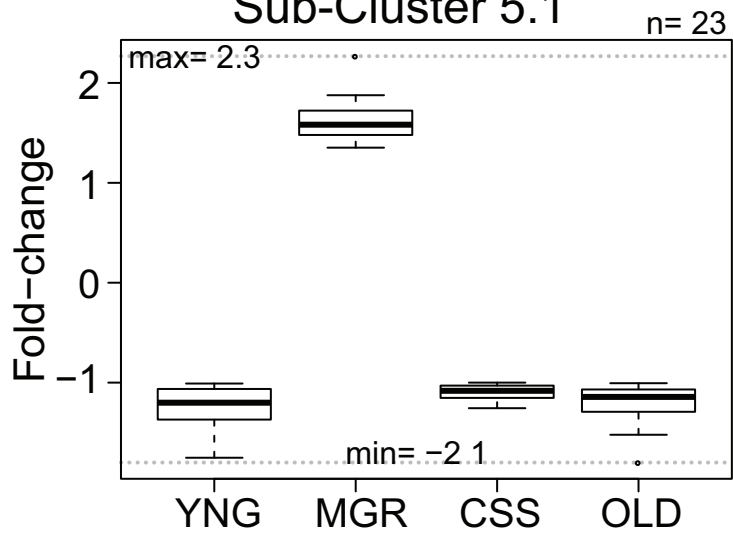

### Sub-Cluster 5.2

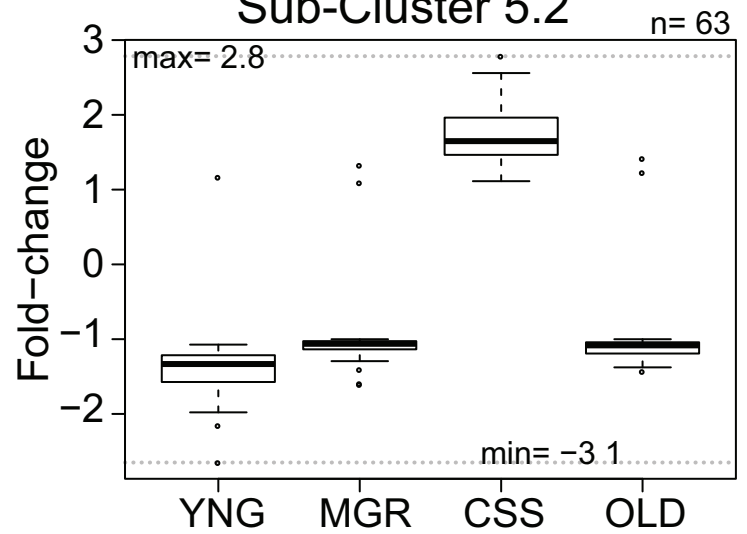

### Cluster8

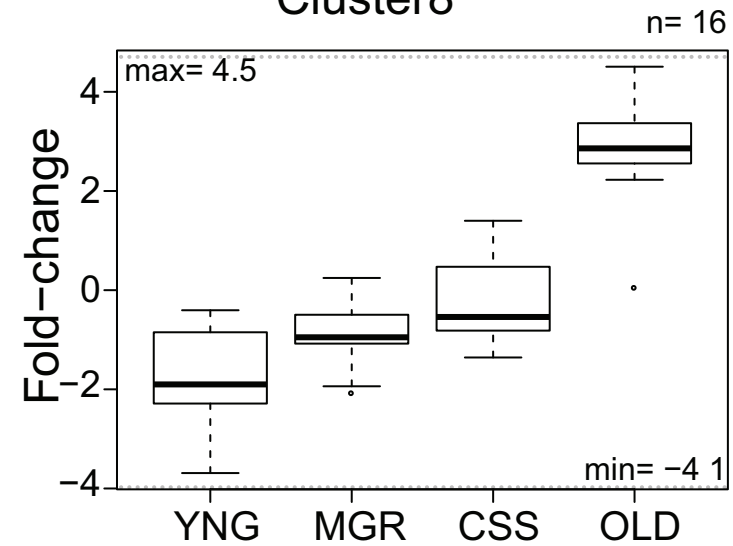

Supplement: Additional file 10: Figure S3 — Gene ontology (GO) SLIM term enrichment analysis for clusters depicted in Figure 2. A) Boxplots depicting distribution of term enrichment across all clusters, expressed as fold-change relative to abundance in the full genome, for each of the three GO SLIM categories; 'cellular component', 'molecular function', and 'biological process'. Boxes bound upper and lower quartiles, dark horizontal bars denote median values, whiskers represent 95% confidence intervals, circles represent outliers occuring in upper and lower 2.5 percentiles. B) Barplots exhibiting term enrichment for each cluster in each of the three GO SLIM categories; colour assignment for bars is indicated in Figure 'A'. The number of genes (accessions) included in each cluster is indicated at the base of the 'biological process' barplot. [file 1471-2229-13-14-S10.pdf]
