# Supplementary material for: Transcriptional programming during cell wall maturation in the expanding Arabidopsis stem
Source: BMC Plant Biol. 2013 Jan 25;13:14. doi: 10.1186/1471-2229-13-14 (PMC3635874; doi:10.1186/1471-2229-13-14)
Supplement: Additional file 1: Figure S1 — Surface plots of relative elongation growth rates and LOWESS-predicted growth kinematic profiles (n=34). Plotting as described in Hall & Ellis (2012) except that segments are numbered from bottom upwards for surface plots. Plants arranged by column according to independently grown and observed batches. [file 1471-2229-13-14-S1.pdf]

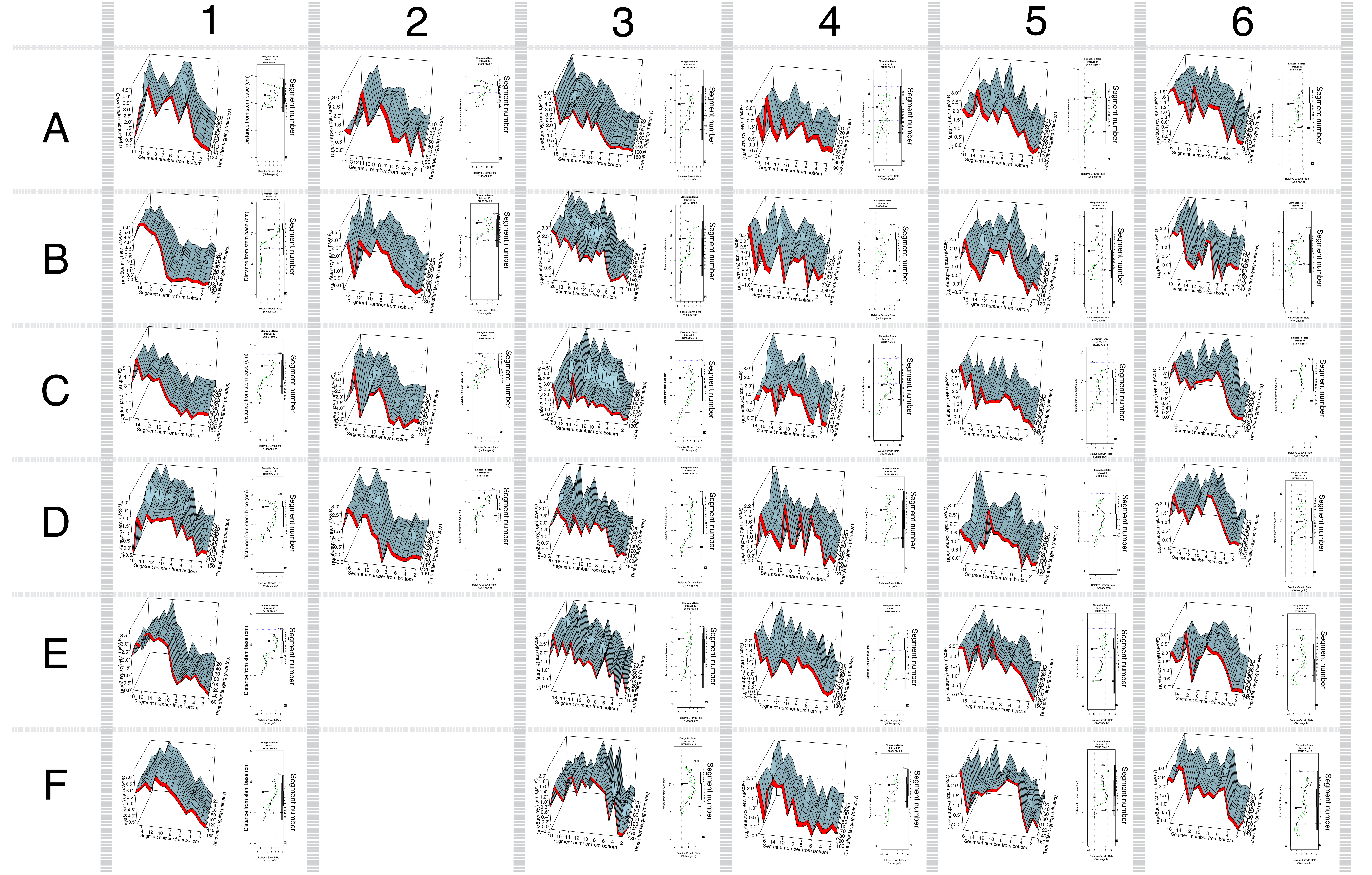
1
2
3
4
5
6
A
B
C
D
E
F
Growth rate (%change/hr)
Segment number from bottom
Time after tagging (minutes)
Distance from stem base (cm)
Relative Growth Rate (%change/hr)
Segment number
